# Development and preclinical evaluation of novel fluorinated ammonium salts for PET myocardial perfusion imaging

**DOI:** 10.1038/s41598-021-99212-0

**Published:** 2021-10-04

**Authors:** Ofer Shamni, Hilbert Grievink, Netanel Kolevzon, Seweryn Krajewski, Lukasz Steczek, Ella Meltzer, Shimon Yitshak, Eyal Mishani, Galith Abourbeh

**Affiliations:** 1 Cyclotron/Radiochemistry/MicroPET Unit, Hadassah Medical Organization and Faculty of Medicine, Hebrew University of Jerusalem, 91120 Jerusalem, Israel; 2 Synektik LTD, Warsaw, Poland

**Keywords:** Cardiology, Nuclear chemistry, Preclinical research

## Abstract

We previously presented the radiolabeled ammonium salt [^11^C]-dimethyl diphenylammonium trifluoromethanesulfonate ([^11^C]DMDPA) as a potential novel PET-MPI agent. The current study aimed to increase the clinical applicability of PET-MPI by designing and synthesizing fluorinated ammonium salt derivatives. Four fluorinated DMDPA derivatives and two quinolinium salt analogs were radiolabeled. The dynamic distribution in vivo, following injection of each derivative into male SD rats, was evaluated using small-animal dedicated PET/CT. Organ uptake after injection of [^18^F]fluoroethylquinolinium acetate ([^18^F]FEtQ) was examined ex vivo. Four fluorinated DMDPA derivatives were synthesized, two were labeled with fluorine-18: [^18^F]fluoroethyl-methyldiphenylammonium trifluoromethanesulfonate ([^18^F]FEMDPA) and [^18^F]fluorobuthyl-methyldiphenylammonium trifluoromethanesulfonate ([^18^F]FBMDPA). The other two were labeled using carbon-11: [^11^C]methyl-(3-fluorophenyl)-methylphenylammonium trifluoromethanesulfonate ([^11^C]3-F-DMDPA) and [^11^C]methyl-(4-fluorophenyl)-methylphenylammonium trifluoromethanesulfonate ([^11^C]4-F-DMDPA). All four DMDPA derivatives exhibited significantly lower heart/liver radioactivity uptake ratios (0.6, 0.4, 0.7 and 0.6, respectively) compared to that of [^11^C]DMDPA (1.2). Conversely, the two radiolabeled quinolinium salt derivatives, [^11^C]methylquinolinium iodide ([^11^C]MeQ) and [^18^F]FEtQ demonstrated improved heart/liver ratios (2.0 and 1.3, respectively) with clear visualization of the left ventricle myocardium. Renal clearance was the major route of elimination. Among the fluorinated quaternary ammonium salts tested, [^18^F]FEtQ yielded the best images. Further studies are in progress to elucidate the underlying mechanism of its cardiac uptake.

## Introduction

Heart disease is the leading cause of death for both men and women in the Western world, with coronary artery disease (CAD) being the most common heart disease^1^. Evaluation of myocardial ischemia and its functional implications are important in the diagnosis, risk assessment, and management of patients with known or suspected CAD. Nuclear myocardial perfusion imaging (MPI) using single-photon emission computed tomography (SPECT), and to a lesser extent, positron emission tomography (PET), are the prevailing non-invasive approaches for determining the presence and extent of coronary stenosis. Quantitative MPI has been demonstrated using SPECT, cardiac magnetic resonance imaging, and computed tomography perfusion. Nevertheless, PET remains the gold standard for the non-invasive quantification of myocardial blood flow (MBF) and coronary flow reserve (CFR)^2^. Both MBF and CFR have been established as useful measures for diagnosing or ruling out obstructive CAD, for risk stratification, and patient management^3,4^.

Rubidium-82, ^13^N-ammonia, and ^15^O-water are well-validated PET radiopharmaceuticals for quantitative MPI. The short half-lives of oxygen-15 (~ 2 min) and nitrogen-13 (~ 10 min) limit their use to centers with a nearby cyclotron, whereas the cost of ^82^Sr/^82^Rb generators often restricts the use of ^82^Rb to high volume centers. The American Society of Nuclear Cardiology and the Society of Nuclear Medicine and Molecular Imaging have noted significant underutilization of PET-MPI, relative to its demonstrated advantages, for patients being assessed for suspected clinically significant CAD, and to its wide accessibility in the U.S.^5^. The availability of a suitable fluorine-18 labeled pharmaceutical is expected to enhance the use of PET for MPI due to its ideal imaging properties and convenient half-life (~ 110 min), which allows distribution to remote sites. Numerous ^18^F-labeled pharmaceuticals have been investigated for potential PET-MPI use^6^. The most promising agent thus far, [^18^F]flurpiridaz, is currently under a second phase-3 clinical trial, which is predicted to be completed by the end of 2021^7^. [^18^F]FBnTP has also been reported to yield good cardiac images in the myocardium of dogs^8^. Yet, its routine production poses a challenge, as its radiolabeling is a complex multi-step synthesis, and the strength of the final product should be limited owing to radiolysis^9^.

We have previously reported various ^11^C-labeled ammonium salt derivatives for PET cardiac imaging. The lead compound, [^11^C]dimethyl-diphenylammonium trifluoromethanesulfonate ([^11^C]DMDPA), displayed a rapid, high, and prolonged cardiac uptake in mice, rats, and pigs. It also demonstrated high stability in vivo and favorable distribution kinetics with respect to the blood and adjacent non-target organs, such as the liver and lungs^10,11^.

**Figure 1:**
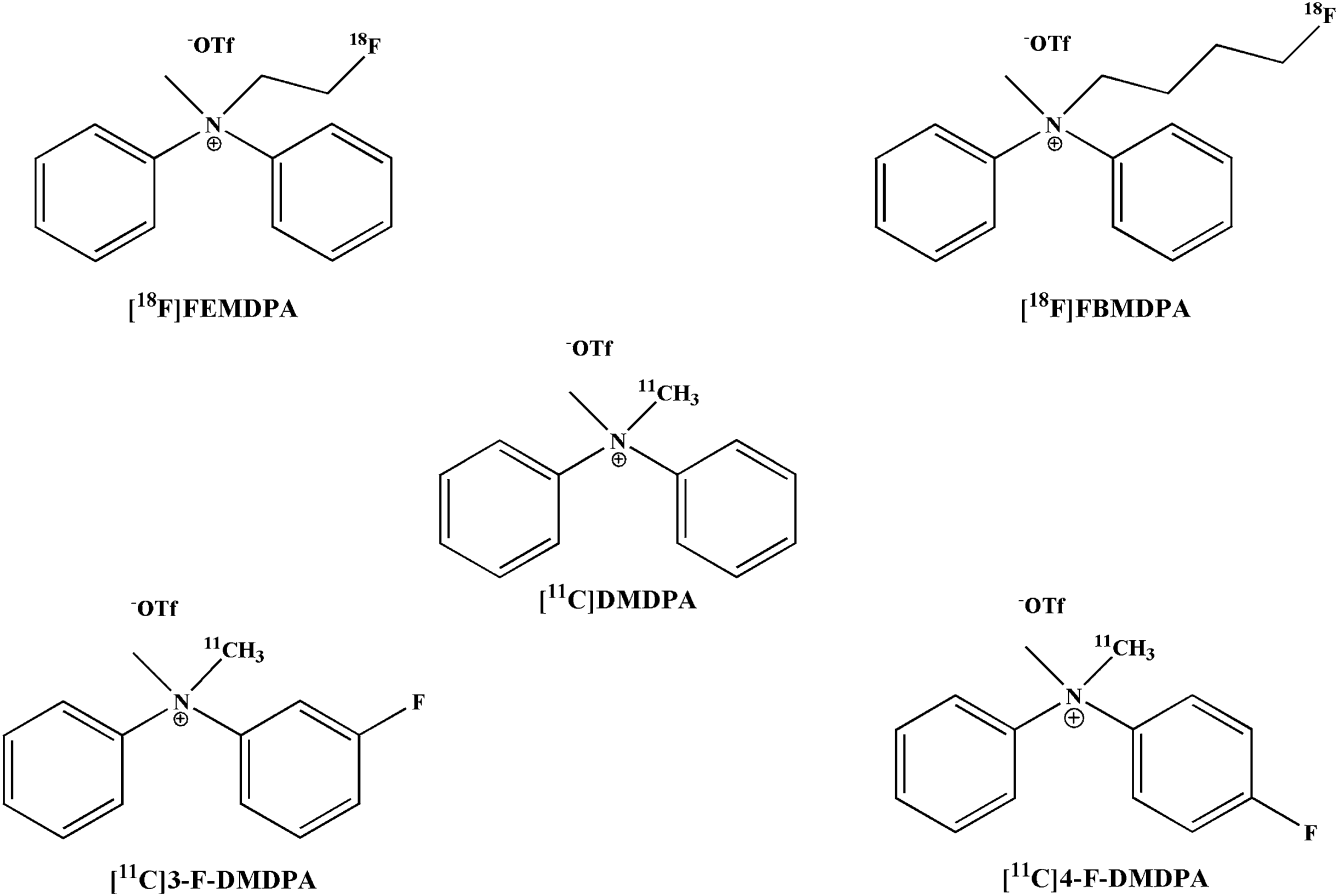
Chemical structures of ^11^C-dimethyl diphenylammonium trifluoromethanesulfonate ([^11^C]DMDPA) and four fluorinated DMDPA-based derivatives*:* 2-[^18^F]fluoroethyl-methyl-diphenylammonium trifluoromethanesulfonate ([^18^F]FEMDPA), [^18^F]4-fluorobutyl-methyl-diphenylammonium trifluoromethanesulfonate ([^18^F]FBMDPA), [^11^C]methyl-(3-fluorophenyl)-methylphenylammonium trifluoromethanesulfonate ([^11^C]3-F-DMDPA) and [^11^C]methyl-(4-fluorophenyl)-methylphenylammonium trifluoromethanesulfonate ([^11^C]4-F-DMDPA).

**Figure 2:**
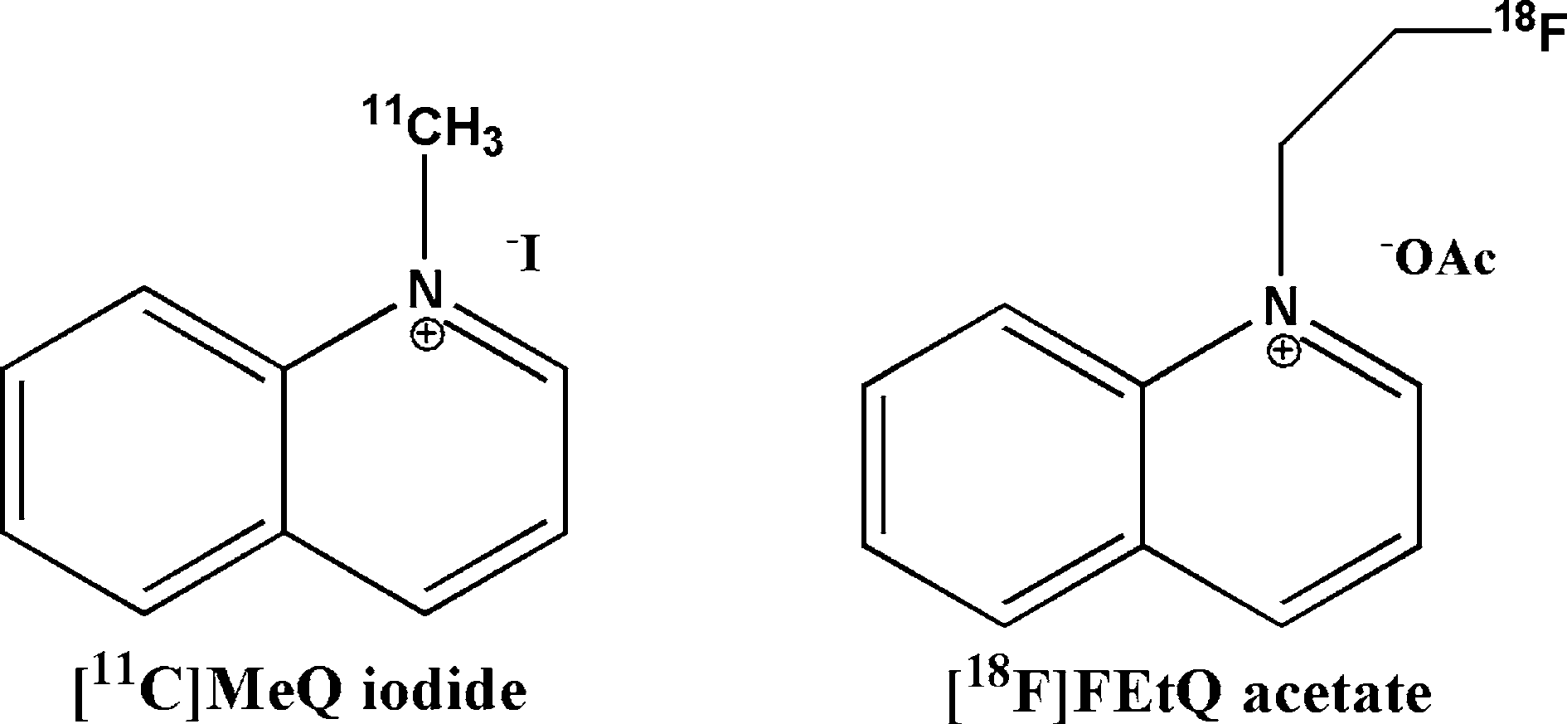
Chemical structures of [^11^C]methylquinolinium iodide ([^11^C]MEtQ) and [^18^F]fluoroethylquinolinium acetate ([^18^F]FEtQ).

In the present study, the evaluation of ammonium salts as potential PET-MPI probes was extended to fluorinated derivatives of DMDPA and two additional quinolinium analogs (Figs. 1 and 2). Here we report their reference standard syntheses, radiosyntheses, and the distribution kinetics, following injection of the radiolabeled compounds into male SD rats, as evaluated by small-animal dedicated PET/CT.

## Materials and methods

### Chemistry and radiochemistry

The conditions for the cyclotron production of carbon-11 and fluoride-18, used for radiolabeling of the various synthesized derivatives, are described below. The corresponding radiochemistry and chemistry of the non-radiolabeled reference standards and precursors are described in the Supplementary Material.

#### Carbon-11 production

Carbon-11 CO_2_ was produced on an 18/9 IBA cyclotron, by the ^14^N(p,α)^11^C nuclear reaction, using nitrogen containing 0.5% oxygen, as previously described^12^. At the end of bombardment, the target gas was delivered and trapped by a cryogenic trap. Radiolabeling was performed by fully automated processes, using a [^11^C]CH_3_I module (G.E. Medical Systems, Münster, Germany), to yield the desired ^11^C-labeled derivative.

#### ^*18*^*F-fluoride production*

Fluoride-18 anion was produced via the ^18^O(p,n)^18^F nuclear reaction using an 18/9 IBA cyclotron equipped with a target for generating ^18^F-fluoride, as previously described^13^. ^18^F-fluoride was delivered from the cyclotron (in 3 mL of [^18^O]H_2_O) and trapped using a solid-phase extraction (SPE) anion-exchange cartridge (Chromafix 30PS-HCO_3_, Marcherey Nagle, Düren, Germany). Radiolabeling was performed by fully automated processes, using a GE FxFN module (G.E. Medical Systems, Münster, Germany).

### Biology

Sprague Dawley (SD) rats (male, 283–323 g) were obtained from Envigo (Rehovot, Israel). All animal studies were approved by the Animal Research Ethics Committee of the Hebrew University of Jerusalem (ethical approval number MD-13-13664-3) and conducted following its guidelines. Reporting was carried out in accordance with the ARRIVE guidelines. Animals were allowed to acclimate for at least 3 days before the imaging studies, routinely kept in 12 h light/dark cycles, and provided with food and water ad libitum.

#### PET/CT acquisitions and image analysis

PET/CT acquisitions and image analyses were conducted as previously described^13^. Male SD rats were anesthetized with isoflurane (2.5% in O_2_) and maintained normothermic using a heating pad. Following a CT attenuation-correction scan, PET acquisitions were carried out in list-mode using an Inveon™ MM PET-CT small animal-dedicated scanner (Siemens Medical Solutions, USA). PET scans were started at tracer injection and lasted for 25–60 min, depending on the injected radiopharmaceutical^13^.

Emission sinograms were normalized and corrected for attenuation, scatter, randoms, dead time, and decay. The scans were divided into the following time frames: 6 × 10 s, 8 × 30 s, 5 × 60 s, 3 × 300 s ([^11^C]DMDPA, [^11^C]3-F-DMDPA, [^11^C]4-F-DMDPA and [^11^C]MeQ), 6 × 10 s, 8 × 30 s, 5 × 60 s, 10 × 300 s ([^18^F]FEMDPA and [^18^F]FBMDPA) and 6 × 10 s, 8 × 30 s, 5 × 60 s, 5 × 300, 1 × 600 s ([^18^F]FEtQ). Image reconstruction was performed using Fourier rebinning and two-dimensional ordered-subsets expectation maximization (2D-OSEM), with a voxel size of 0.776 × 0.776 × 0.796 mm^3^. Image analysis and quantification were performed using the Inveon Research Workplace (IRW) 4.2 (Siemens). Delineation of volumes of interest (VOIs) was performed by manual segmentation, based on the PET and CT images, and the corresponding time-activity curves (TACs) were calculated. Delineation of the LV myocardium and the blood-pool was done using the automated rodent cardiac segmentation tool of the IRW software, followed by a 1- and 2-mm erosion of the resulting VOIs of the LV myocardium and the blood-pool, respectively. The distribution of radioactivity was calculated as the percentage of injected dose per mL of tissue (%ID/mL). Standardized uptake values (SUVs) were calculated as the product of %ID/mL and the total body weight of the animal^13^. Unless otherwise stated, the reported time-point for each SUV represents the mid time-frame.

#### Ex vivo biodistribution studies of [^18^F]fluoroethylquinolinium acetate ([^18^F]FEtQ)

At the end of each 45-min [^18^F]FEtQ PET acquisition, rats were sacrificed by an i.p. injection of pentobarbitone sodium (CTS chemical industries Ltd., Kiryat Malachi, Israel). Organs and tissues of interest were excised, rinsed in PBS, blotted and weighed, and their radioactivity content was measured using a gamma counter (2480 Wizard2, PerkinElmer, MA, USA). The presence of radioactive metabolites in the urine was analyzed by injecting 100 µL of urine samples into an analytical HPLC, as described for the radiolabeling of [^18^F]FEtQ (Supplementary Material).

## Results

### PET/CT studies of fluorinated DMDPA derivatives

#### *[*^*18*^*F]fluoroethyl-methyldiphenylammonium trifluoromethanesulfonate ([*^*18*^*F]FEMDPA) and [*^*18*^*F]fluorobuthyl-methyldiphenylammonium trifluoromethanesulfonate [*^*18*^*F]FBMDPA*

**Figure 3:**
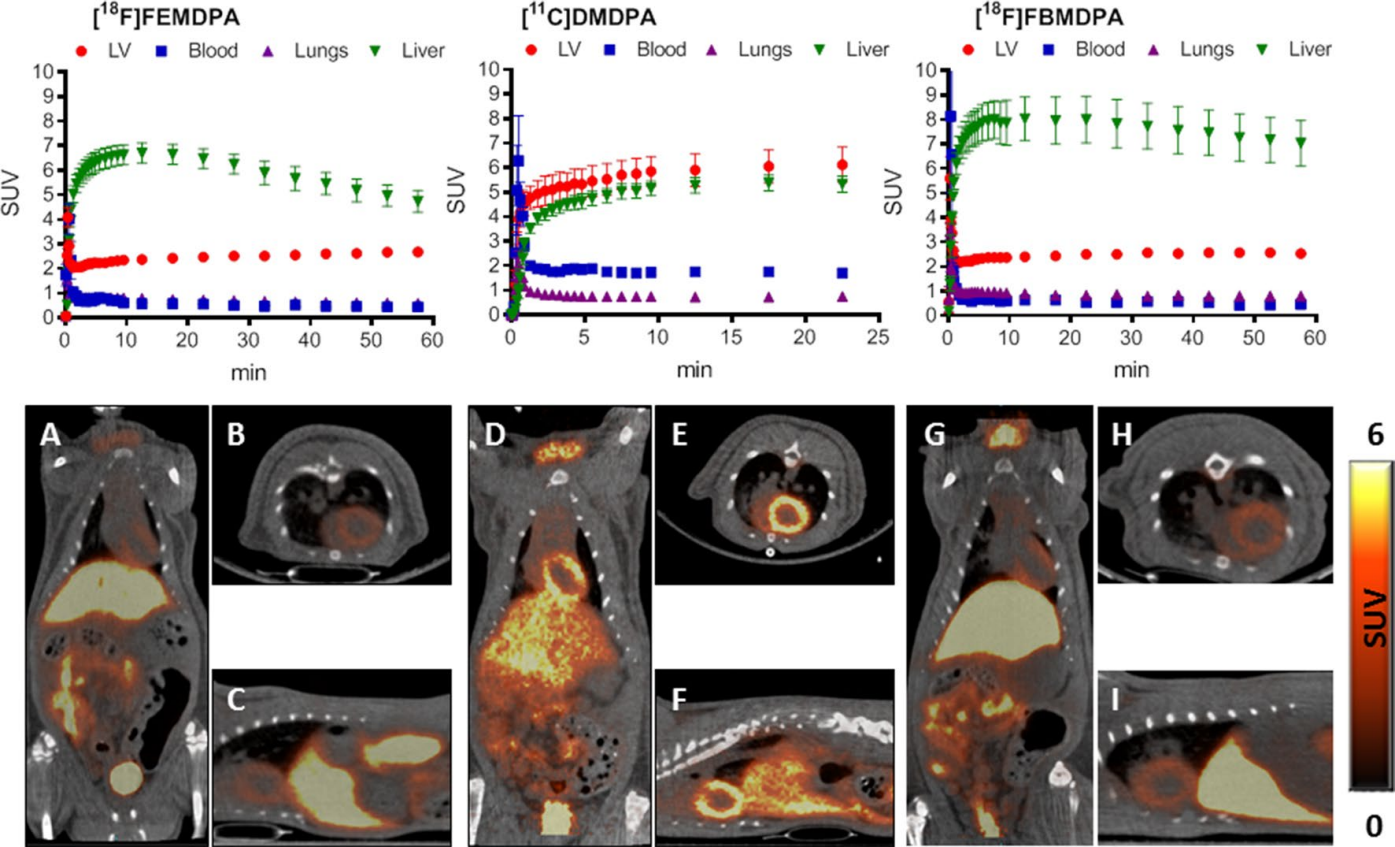
TACs of selected organs following i.v. injection of [^18^F]FEMDPA (upper left, n = 4), [^11^C]DMDPA (upper middle, n = 4) and [^18^F]FBMDPA (upper right, n = 3) into male SD rats. Results are presented as mean ± SEM. Representative PET/CT of coronal (**A**,**D**,**G**), axial (**B**,**E**,**H**), and sagittal (**C**,**F**,**I**) slice images obtained following i.v. injection of [^18^F] FEMDPA (**A**–**C**), [^11^C]DMDPA (**D**–**F**) and [^18^F]FBMDPA (**G**–**I**), into male SD rats. Images represent the summation of 15–25 min frames.

Following i.v. injection of [^18^F]FEMDPA (20.2 ± 7.8 MBq, n = 4) and [^18^F]FBMDPA (21.7 ± 6.7 MBq, n = 3) into rats, both compounds exhibited rapid accumulation of radioactivity in the left ventricle (LV) myocardium and the liver (Fig. 3). These findings are in agreement with the rapid uptake of [^11^C]DMDPA in these organs. As with [^11^C]DMDPA, both fluorinated compounds also displayed rapid washout from the lungs and blood pool. However, the uptake of [^18^F]FEMDPA and [^18^F]FBMDPA was higher in the liver and lower (two–threefold) in the LV myocardium, compared to [^11^C]DMDPA. Owing to the moderate washout of [^18^F]FEMDPA and [^18^F]FBMDPA from the liver, peak heart/liver radioactivity uptake ratios of 0.57 and 0.36 were measured 60 min after injection, suggesting that these compounds are less favorable than [^11^C]DMDPA for cardiac imaging (Fig. 3).

#### *[*^*11*^*C]methyl-(3-fluorophenyl)-methylphenylammonium trifluoromethanesulfonate ([*^*11*^*C]3-F-DMDPA)*

**Figure 4:**
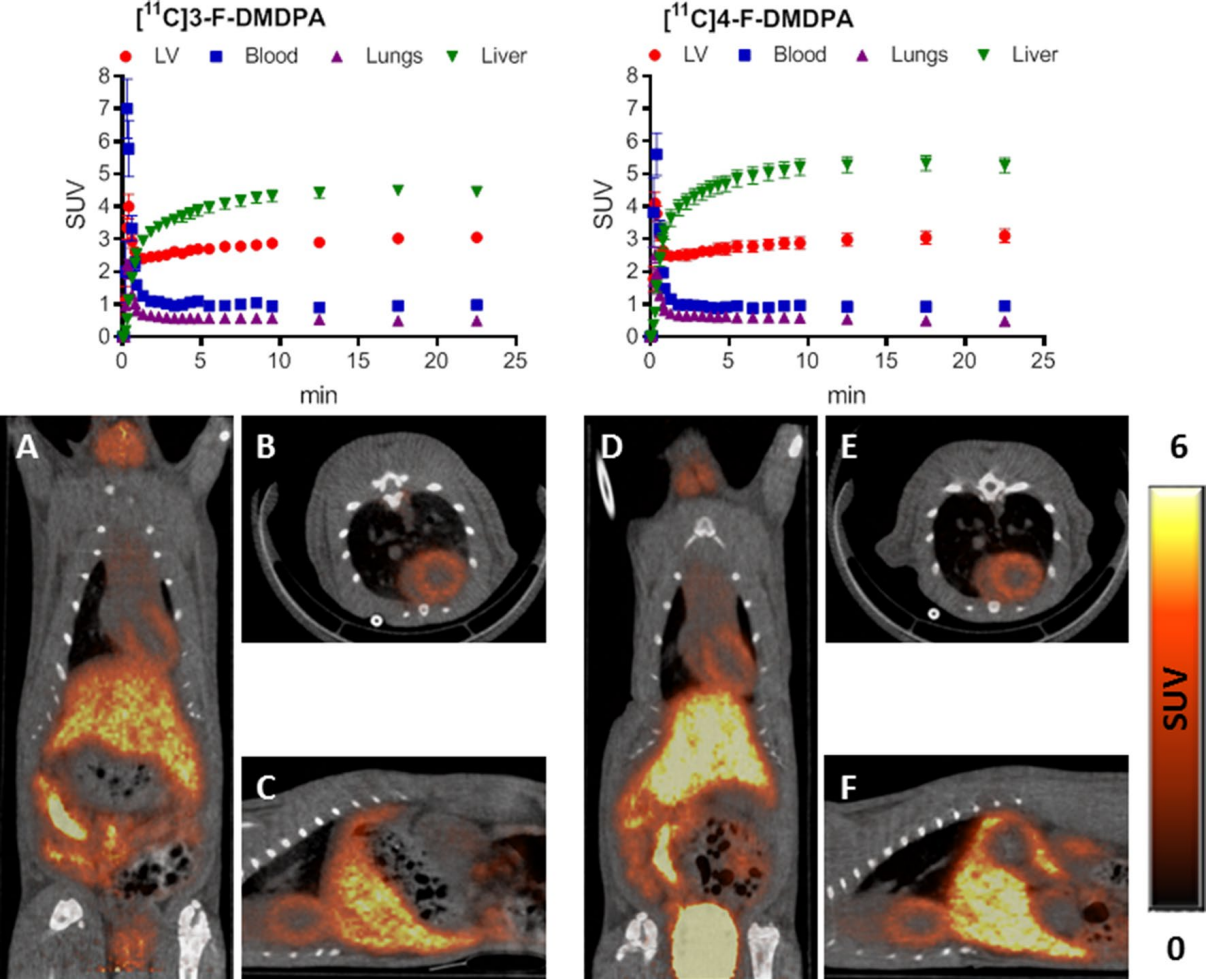
TACs of selected organs following i.v. injection of [^11^C]3-F-DMDPA (upper left, n = 5) and [^11^C]4-F-DMDPA (upper right, n = 6) into male SD rats. Results are presented as mean ± SEM. Representative PET/CT of coronal (**A**,**D**), axial (**B**,**E**), and sagittal (**C**,**F**) slice images obtained following i.v. injection of [^11^C]3-F-DMDPA (**A**–**C**) and [^11^C]4-F-DMDPA (**D**–**F**) into male SD rats. Images represent the summation of 15–25 min frames.

Following i.v. injection of [^11^C]3-F-DMDPA (15.6 ± 1.2 MBq, n = 5) into rats, rapid accumulation of radioactivity was observed in the LV myocardium and the liver, with a heart/liver radioactivity uptake ratio of 0.69, which remained constant during the 25 min acquisition (Fig. 4, right). Compared to its non-fluorinated analog, [^11^C]DMDPA, [^11^C]3-F-DMDPA had a two-fold lower cardiac SUV (3.1 vs. 6.1 at 25 min). Yet, its liver SUV was only 15% lower than that of [^11^C]DMDPA (4.5 vs. 5.3 at 25 min), resulting in a less favorable profile for cardiac imaging (Fig. 4). Both compounds possessed similar characteristics with respect to their distribution in other organs, namely rapid washout from the blood and the lungs and high renal clearance (data not shown).

#### *[*^*11*^*C]methyl-(4-fluorophenyl)-methylphenylammonium trifluoromethanesulfonate ([*^*11*^*C]4-F-DMDPA)*

The TACs obtained following i.v. injection of [^11^C]4-F-DMDPA (16.2 ± 0.8 MBq, n = 6) into rats are presented in Fig. 4, revealing a distribution profile similar to that of [^11^C]3-F-DMDPA, yet with a more pronounced accumulation in the liver, resulting in a heart/liver radioactivity uptake ratio of 0.58. Thus, although the LV myocardium could be visualized following administration of [^11^C]4-F-DMDPA (Fig. 4, left), its relatively high accumulation in the liver renders it a less suitable candidate for cardiac imaging compared to [^11^C]DMDPA.

### PET/CT studies of quinolinium analogs

#### *[*^*11*^*C]methylquinolinium iodide ([*^*11*^*C]MeQ)*

**Figure 5:**
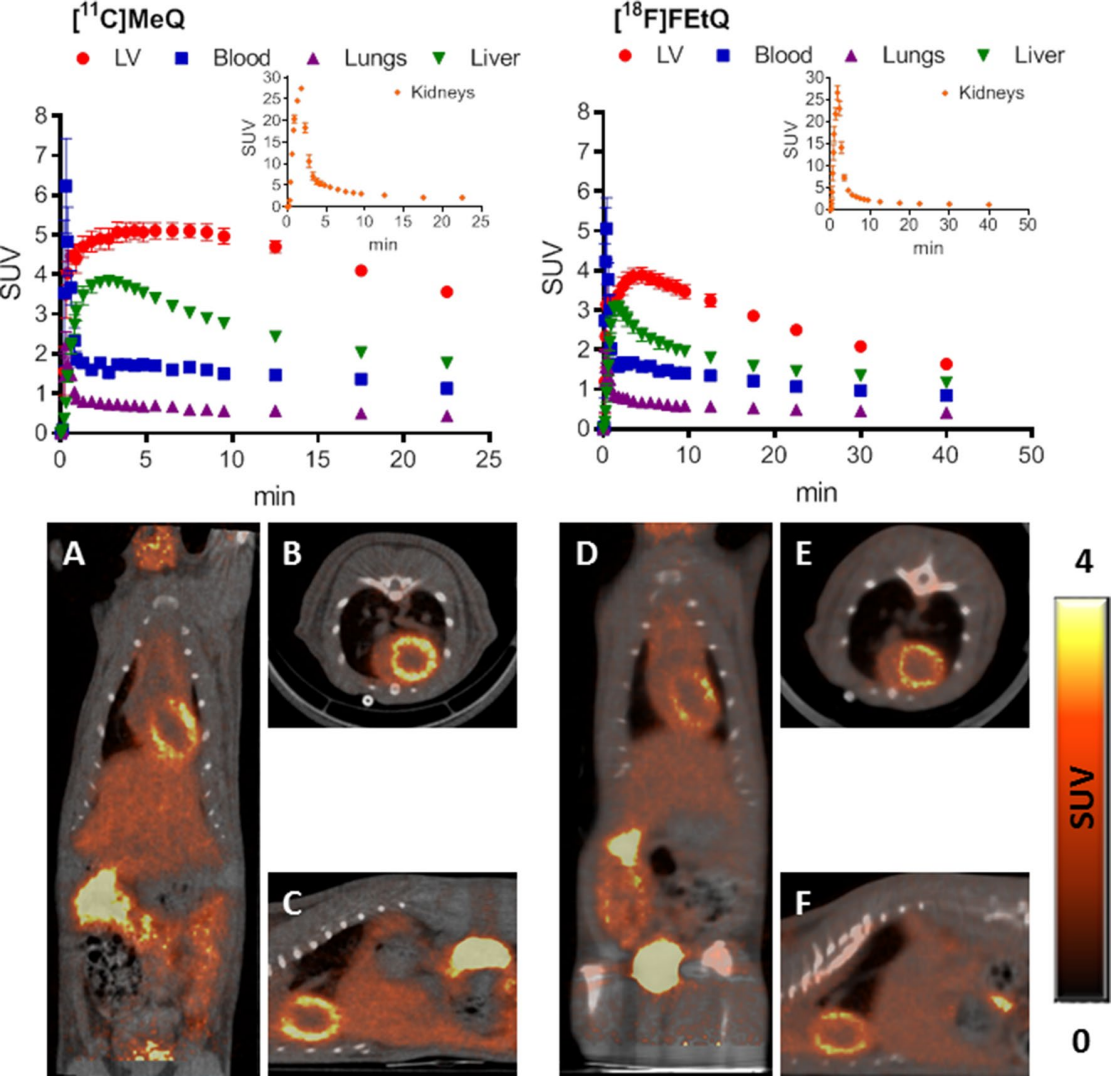
TACs of selected organs following i.v. injection of [^11^C]MeQ (left, n = 3), and [^18^F]FEtQ (right, n = 7) into male SD rats. Insets: distribution kinetics in the kidneys. Results are presented as mean ± SEM. Representative PET/CT of coronal (**A**,**D**), axial (**B**,**E**), and sagittal (**C**,**F**) slice images obtained following i.v. injection of [^11^C]MeQ (**A**–**C**) and [^18^F]FEtQ (**D**–**F**) into male SD rats. Images represent the summation of 15–25 min frames.

The TACs presented in Fig. 5 depict the distribution of [^11^C]MeQ following i.v. injection to rats (15.9 ± 1.0 MBq, n = 3), illustrating rapid LV myocardium uptake of the compound, with only moderate (~ 25%) washout within 25 min. Within 5 min from injection, the radioactivity uptake ratio between the heart and the blood, the lung and the liver was ≥ 3.0, 7.3 and 1.5, respectively. Consequently, the LV myocardium could be clearly visualized, as illustrated in Fig. 5A–C. Similar to the above-mentioned quaternary ammonium salts, [^11^C]MeQ exhibited high renal clearance.

#### *[*^*18*^*F]fluoroethylquinolinium acetate ([*^*18*^*F]FEtQ)*

The TACs presented in Fig. 5 were obtained following i.v. injection of [^18^F]FEtQ (17.7 ± 1.3 MBq, n = 7) into rats, illustrating rapid radioactivity uptake in the LV myocardium and the liver, followed by pronounced washout from both organs. Consequently, at 45 min after injection, radioactivity levels in the LV myocardium and the liver were only approximately 40% of those measured at the peak of accumulation. The observed washout of radioactivity from the LV myocardium and the liver is distinguishable from the high and prolonged accumulation of [^11^C]DMDPA in these organs (Figs. 3 and 4). Thus, the measured cardiac and liver SUVs at 25 min after injection of [^18^F]FEtQ were only 2.5 and 1.5, respectively, compared to 5.9 and 4.9 after injection of [^11^C]DMDPA. Nonetheless, the LV myocardium could be clearly visualized (Fig. 5D–F), and at 45 min after injection, the radioactivity uptake ratio between the heart and the blood, the lung and the liver was 1.9, 4.0 and 1.4, respectively.

### Ex vivo distribution of [^18^F]FEtQ

Rats were sacrificed following PET acquisition to study the distribution of radioactivity 45 min after injection of [^18^F]FEtQ. Organs and tissues of interest were harvested, and radioactivity concentrations were measured. The ex vivo distribution results presented in Table 1 were in good agreement with those obtained from the analyses of PET images. As expected from a cationic quaternary salt, the primary route of elimination was via renal clearance. Except for the urinary bladder, the highest radioactivity concentrations were measured in the heart, followed by the kidneys and the liver.

**Table 1:** Comparison of SUVs calculated from ex vivo distribution studies and PET images at 45 min and from a 35–45 min time interval after injection of [^18^F]FEtQ, respectively.

Tissue/organ	SUV (Biodistribution)	SUV (PET)
Heart	1.7 ± 0.3	1.6 ± 0.3
Lungs	0.6 ± 0.1	0.4 ± 0.1
Thymus*	0.8 ± 0.4	0.9 ± 0.4
Liver	1.0 ± 0.1	1.2 ± 0.1
Spleen	0.4 ± 0.1	ND
Right kidney	1.5 ± 0.2	1.2 ± 0.2
Left kidney	1.5 ± 0.2	1.2 ± 0.2
Muscle	0.4 ± 0.0	0.5 ± 0.1
Bone	0.6 ± 0.1	0.6 ± 0.1
Skin*	0.4 ± 0.1	ND
Urine	76.8 ± 30.2	ND
Blood	0.6 ± 0.1	0.8 ± 0.2

Results are presented as mean ± S.D (n = 7; in organs marked with an asterisk n = 3). ND, not determined.

The results of metabolite analysis in the urine, presented in Table 2, indicate that 45 min after i.v. injection of [^18^F]FEtQ, most of the radioactivity could be attributed to the intact compound, which comprised 77 ± 10% (n = 7) of the total radioactivity in the urine.

**Table 2:** Results of HPLC analysis of urine samples 45 min after i.v. injection of [^18^F]FEtQ.

Rat #	Percentage of intact [^18^F]FEtQ in urine samples 45 min after i.v. injection
1	84.5
2	81.1
3	80.3
4	74.4
5	89.0
6	71.1
7	60.0

## Discussion

Over the past decade, our group has investigated the potential use of radiolabeled ammonium salt derivatives for PET-MPI^10–12^. The previously reported lead compound, [^11^C]DMDPA, yielded good quality images with a more favourable distribution pattern and/or enhanced heart/tissue radioactivity uptake ratios, compared to established MPI probes, such as [^99m^Tc]MIBI and [^13^N]NH_3_, in mice, rats and pigs^10,11^. Metabolite analysis of blood, liver and urine samples following injection of [^11^C]DMDPA into rats indicated the compound was metabolically stable. The performance of [^11^C]DMDPA under changes in blood flow induced by adenosine stress was also reported^10^ and was comparable to the results acquired with [^13^N]NH_3_, under the same conditions. Moderate effect of isoflurane anesthesia on the biodistribution of [^11^C]DMDPA was also demonstrated^10^ and was in line with previous reports^14–16^. Moreover, it was found safe for human administration at the relevant clinical dosages, as indicated by rat toxicity studies (data not shown).

To increase the clinical applicability of ammonium salts, the current study focused on developing fluorine-18 labeled quaternary ammonium derivatives, relying initially on DMDPA-based structures. Four fluorinated DMDPA derivatives (Fig. 1) were designed, synthesized, and evaluated in vivo using two approaches for the radiolabeling. The first approach entailed an aliphatic nucleophilic substitution at the quaternary nitrogen, substituting one methyl group in DMDPA with either fluoroethyl (to yield [^18^F]FEMDPA) or fluorobutyl (to yield [^18^F]FBMDPA). The second approach entailed ^11^C-methylation of fluoroaryl derivatives at either position 3 or 4 of one aromatic ring, yielding [^11^C]3-F-DMDPA or [^11^C]4-F-DMDPA, respectively. In the first approach, short alkyl chains were selected to retain the structure of DMDPA as much as possible. Various synthetic procedures were initially undertaken to synthesize 1-fluoromethyl-methyldiphenylammonium, yet without success (data not shown). Subsequently, [^18^F]FEMDPA and [^18^F]FBMDPA were designed and successfully synthesized via N-aliphatic alkylation. Both compounds were prepared by a fully automated synthesis, using a GE TRACERlab FxFN module (Schemes 7s and 8s). However, both derivatives exhibited less favorable profiles for cardiac imaging, as reflected by reduced LV myocardium uptake and increased liver accumulation compared to [^11^C]DMDPA (Fig. 3). Furthermore, attempts were made to synthesize ^18^F-fluoroaryl DMDPA analogues (i.e. [^18^F]-3-F-(aryl)-DMDPA and [^18^F]-4-F-(aryl)-DMDPA), yet the radiochemical yields and radiochemical purities were low (unpublished data). Subsequently, an alternative approach, which circumvents the challenging fluorine-18 labeling, was chosen to assess the potential of the two aryl-fluoride DMDPA derivatives. The corresponding non-radiolabeled fluorinated-diphenylamine precursors were used as starting materials and labeled with carbon-11, yielding [^11^C]3-F-DMDPA and [^11^C]4-F-DMDPA. Still, rat imaging studies suggested the two derivatives presented less favorable profiles for cardiac imaging, with approximately twofold lower heart/liver radioactivity uptake ratios, compared to that of [^11^C]DMDPA (Figs. 3 and 4).

These results indicate that despite the relatively minor structural modifications, compared to [^11^C]DMDPA, all four fluorinated DMDPA derivatives performed less favorably vis-à-vis their potential for cardiac imaging. It is hypothesized that such differences could be attributed to the relatively high electronegativity of the fluorine atom and the changes it inflicts to the overall polarity and charge density of a small molecule like DMDPA. Further studies are required to corroborate this.

In light of these findings, we moved forward and investigated a different quaternary ammonium cation scaffold, i.e. quinolinium salts (Fig. 2). We initially radiolabeled the quinoline using one [^11^C]-methylation (Scheme 9s) to allow a preliminary assessment of its myocardial imaging properties. In vivo imaging following its i.v. injection to rats resulted in clear visualization of the LV myocardium and a significant improvement in the heart/liver radioactivity uptake ratio compared to [^11^C]DMDPA (2.0 vs. 1.2) (Fig. 5A–C). These results encouraged us to further develop a fluorine-18-labeled quinolinium derivative. The fluorinated non-radiolabeled, fluoroethyl quinolinium reference standard was prepared using fluoroethyl tosylate (Scheme 5s) in a slow kinetic (48 h) synthesis (supplementary data). The radiolabeling of the corresponding [^18^F]FEtQ, was performed by a fully automated one-step radiosynthesis using ethyltrifluoromethanesulfonate quinolinium as precursor (Scheme 10s), in a 44 min process, including HPLC purification. Overall, 13.3 ± 4.3 GBq was obtained (n = 13), with a radiochemical purity greater than 99% and a radiochemical yield of 14.4 ± 4.3% D.C. to EOB. Following injection of [^18^F]FEtQ into rats, the LV myocardium could be visualized (Fig. 5D–F), with a heart/liver radioactivity uptake ratio of 1.3 and 1.7 at 45 min after injection, as measured by PET imaging and ex vivo biodistribution, respectively. In line with [^11^C]DMDPA and the other investigated quaternary ammonium salts, [^18^F]FEtQ exhibited rapid washout from the blood and high renal clearance. Consequently, the stability of [^18^F]FEtQ in vivo was investigated using urine samples. Analysis of the radioactivity present in urine at 45 min after injection revealed that 77 ± 10% could be attributed to the intact compound, indicating high stability in vivo. Future studies will need to elucidate if [^18^F]FEtQ, like [^11^C]DMDPA, is also stable in the blood, liver, and myocardium^10,11^.

An investigation of the mechanism underlying the myocardial uptake of quaternary ammonium salts, such as [^18^F]FEtQ, and a direct comparison to additional MPI tracers (e.g. flupiridaz) in various species, was beyond the scope of the current study. Future studies are in progress to address these issues and elucidate the possible involvement of novel and/or established myocardial uptake mechanisms.

## Conclusions

Four novel fluorinated DMDPA derivatives were synthesized and evaluated in vivo. Albeit the relatively modest modifications in the DMDPA scaffold, all four compounds demonstrated significantly lower cardiac accumulation than [^11^C]DMDPA, rendering them less favorable candidates for cardiac imaging. Conversely, clear visualization of the LV myocardium could be demonstrated following injection of the quinolinium derivative [^18^F]FEtQ into rats, with a heart/liver radioactivity uptake ratio on a par with that of [^11^C]DMDPA. Future studies are warranted to further investigate the mechanism of cardiac uptake and the potential of [^18^F]FEtQ as a PET-MPI agent.

## Supplementary Information

Supplementary Information 1.
