# Supplementary material for: Development and preclinical evaluation of novel fluorinated ammonium salts for PET myocardial perfusion imaging
Source: Sci Rep. 2021 Oct 4;11:19693. doi: 10.1038/s41598-021-99212-0 (PMC8490395; doi:10.1038/s41598-021-99212-0)
Supplement: Supplementary file 1 — Supplementary Information 1. [file 41598_2021_99212_MOESM1_ESM.docx]

**Supplementary Material**

*Chemistry of DMDPA-based derivatives*

*Dimethyldiphenylammonium trifluoromethansulfonate (DMDPA)*

Synthesis of the DMDPA non-radiolabeled reference standard was previously described ([1](#_ENREF_1),[2](#_ENREF_2)).

*2-fluoroethyldiphenylamine*

Diphenylamine (1.5 g, 8.86 mmol) and sodium hydride (234 mg, 9.75 mmol) were stirred in dry dibutyl ether (20 mL), and the reaction was refluxed at 135°C for 3 h (scheme 1s). The reaction was cooled on an ice bath, and 1-bromo-2-fluoro-ethane (1.12 g, 8.86 mmol) was added dropwise. The reaction was refluxed for an additional 3 h at 120°C, followed by cooling to room temperature. Water (30 mL) was then added, and the aqueous phase was extracted with ethyl acetate. The organic phase was dried using magnesium sulfate, filtered, concentrated, and dissolved using hexane and purified using flash column chromatography and hexane as an eluent to yield 142 mg of yellow solid (7.5%). ^1^H NMR (CDCl_3_): δ 7.27-7.32 (m, 5H), 6.97-7.1 (m, 5H), 4.64 (dt, *J*1 = 6.6 Hz, *J*2 = 47.1 Hz, 2H), 4.07 (dt, *J*1 = 5.4 Hz, *J*2 = 22.8 Hz, 2H). HRMS 216.11711 (M+1, calculated: 216.11830). Elemental analysis C_15_H_17_NO_3_S: Calculated: C=78.11%, H=6.56%, N=6.51%, F=8.83%. Found: C=78.36%, H=6.72%, N=6.38% F=9.25%.

*2-fluoroethylmethyldiphenylammonium trifluoromethanesulfonate*

2-fluoroethyldiphenylamine (scheme 1s, 130 mg, 0.6 mmol), dissolved in dry dichloroethane (1.5 mL) and methyl trifluoromethanesulfonate (100 µL), was added, after which the reaction was refluxed for 3 h. The product was purified, using flash column chromatography, and 4% methanol in dichloromethane as eluent. ^1^H NMR (DMSO-d_6_): δ 7.56-7.68 (m, 10H), 4.99 (dt, *J*1 = 3.6 Hz, *J*2 = 26.4 Hz, 2H), 4.63 (dt, *J*1 = 3.3 Hz, *J*2 = 48 Hz, 2H), 4.04 (s, 3H). HRMS 230.13252 (M+, calculated: 230.13395). Elemental analysis C_16_H_17_F_4_NO_3_S: Calculated: C=50.66%, H=4.52%, N=3.69%, F=20.03% S=8.45%. Found: C=50.86%, H=4.38%, N=3.58% F=20.50% S=8.69%.

*4-fluorobutyldiphenylamine*

A round bottom flask was loaded with diphenyl-amine (1 g, 5.9 mmol) and NaH (0.18 gr, 7.6 mmol) in 10 mL anhydrous THF (scheme 2s). The reaction mixture was refluxed for 3 h and then cooled in an ice bath. 1-Bromo-4-fluoro-butane (0.83 mL, 7.7 mmol) was added dropwise under nitrogen at room temperature. The reaction was then refluxed for another 16 h. The solvent was evaporated under vacuum and extracted from 50 mL water with DCM (3×50 mL), dried with sodium sulfate, and evaporated again under vacuum. The product was further purified using column chromatography (ethyl acetate/hexanes, 5%: 95%; Rf = 0.94). The final product (scheme 2s) was obtained as a brown oil (98.6 mg, 4.03 mmol, 68.2% yield). ^1^H NMR (300 MHz, CDCl_3_): δ ppm 1.69-1.85 (m, 4 H), 3.752 (t, *J* = 7.5 Hz, 2 H), 4.45 (t, *J* = 47.4, 9 Hz, 2 H), 6.98 (m, 6 H), 7.27 (m, 4 H); ^19^F NMR (300 MHz, CDCl3): δ ppm -217.75 (m, 1 F); HR-MS calculated for C_16_H_18_FN (M + H)+: 244.1502; found 244.1474.

*4-fluorobutylmethyldiphenylammonium trifluoromethansulfonate*

A round bottom flask was loaded with 4-fluorobutyl diphenylamine (200 mg, 0.825 mmol, scheme 2s) in 2 mL anhydrous dichloroethane. Then, methyl trifluoromethanesulfonate (145 µL, 1.32 mmol) was added dropwise under an inert atmosphere at room temperature. The reaction was refluxed for 3 h, cooled, and additional methyl trifluoromethanesulfonate (145 µL, 1.32 mmol) was added (dropwise). The reaction was then refluxed for an additional 2 h. The mixture was subsequently cooled and evaporated under reduced pressure, and purified by chromatography (SiO_2_, ethanol/ dichloroethane, 5%: 95%; Rf = 0.41). The final product was obtained as a brown solid (170.9 mg, 0.66 mmol, 80.2 % yield). ^1^H NMR (300 MHz, CDCl_3_): δ ppm 1.62 (m, 2 H), 1.82-1.91 (m, 2 H), 3.99 (s, 3 H), 4.353 (t, *J* = 5.4 Hz, 1 H), 4.53 (m, 3 H), 7.48-7.61 (m, 10 H); ^19^F NMR (300 MHz, CDCl_3_): δ ppm -220.77 (m, 1 F). HR-MS calculated for C_17_H_21_FN+ (M)+: 258.1658 found 258.1666.

*3-fluorophenyldimethylphenylammonium trifluoromethansulfonate (3-F-DMDPA)*

3-F-DMDPA (Scheme 3s) was obtained by two successive methylation reactions. In the first step, methyl triflate (0.2 mL, 1.77 mmol) was added dropwise under an inert atmosphere, to a solution of 3-fluoro-N-phenylaniline (0.20 g, 1.07 mmol) in anhydrous dichloroethane (10 mL), at room temperature. Subsequently, the reaction was refluxed for 3 h. An additional portion of methyl triflate (0.6 mL, 5.3 mmol) was added at room temperature, and the resulting mixture was refluxed for an additional 3 h. The crude product was evaporated under reduced pressure and purified using silica gel column chromatography and 2% acetone in hexane as eluent, to yield the mono-methylated intermediate (0.15 g, 42% yield). In the second step, methyl triflate (0.6 mL, 5.3 mmol) was added dropwise under an inert atmosphere to a solution of the mono-methylated intermediate (80 mg, 0.40 mmol) in anhydrous dichloroethane (3 mL), at room temperature. The reaction was refluxed for 6 h and then cooled to room temperature. The crude product was evaporated under reduced pressure and purified, using silica gel column chromatography and 10% methanol in dichloroethane as eluent to yield the title compound (70 mg, 78% yield). ^1^H NMR (300 MHz, MeOD-*d*_4_): δ ppm 4.1 (s, 6 H), 7.4-7.69 (m, 9 H); ^19^F NMR (300 MHz, MeOD-*d*_4_): δ ppm -105.8 (s, 1 F); HR-MS calculated for C_14_H_15_FN^+^ (M)^+^: 216.1189, found 216.1180. TLC: Rf = 0.38 (SiO_2_, methanol/DCM, 10%:90%).

*4-fluorophenyldimethylphenylammonium trifluoromethansulfonate (4-F-DMDPA)*

4-F-DMDPA (Scheme 3s) was obtained by two methylation reactions, similar to that of 3-F-DMDPA, except for the 4-fluoro-N-phenylaniline (0.2 g 1.07 mmol) in anhydrous dichloroethane (10 mL), which was used as starting material. The crude product was evaporated under reduced pressure and purified, using silica gel column chromatography and 2% acetone in hexane as an eluent, to obtain the mono-substituted intermediate (Scheme 3s, 0.90 g, 42% yield). In the second step, methyl triflate (0.6 mL, 5.3 mmol) was added dropwise, under an inert atmosphere, to a solution of the mono-methylated (80 mg, 0.41 mmol) in anhydrous dichloroethane (3 mL), at room temperature. The reaction was refluxed for 6 h and then cooled to room temperature. The crude product was evaporated under reduced pressure and purified, using silica gel column chromatography and 10% methanol in methylene chloride as eluent to yield the title compound (70 mg, 78% yield). ^1^H NMR (300 MHz, DMSO-d_6_) δ 4.06 (s, 6H), δ 7.44-7.73 (m, 9H). ^19^F NMR (300 MHz, DMSO-d_6_) δ -106.35 (t, j = 4.2 Hz, 1F). MS (ESI) m/z = 216.1 (M+H^+^).

*Chemistry of quinolinium-based derivatives*

*N-methylquinolinium iodide*

0.55 g of quinoline (Scheme 4s, 4.26 mmol) and 7.98 g (56.2 mmol) of methyl iodide (Sigma Aldrich) were stirred under an inert atmosphere at 40°C overnight. The resulting brown solid was stirred, with a 10 mL mixture of dichloromethane and diethyl ether (1:3), for an additional 1 h and subsequently filtered. The resulting yellow solid was stirred, with a 10 mL mixture of hexane and DCM (6:4), for an additional 1 h. The resulting suspension was filtered to yield the pure product (80% yield). The N-methylquinolinium iodide was analyzed by MS, ^1^H-NMR, HPLC, and TLC. ^1^H NMR (300 MHz, CDCl3) δ 10.21 (d, *J* = 5.7 Hz, 1H), 8.99 (d, *J* = 8.4 Hz, 1H), 8.39 – 8.24 (m, 2H), 8.16 (dd, *J*1 = 8.4 Hz, *J*2= 5.7 Hz, 1H), 8.05 (t, 1H), 4.95 (s, 3H). ESI-MS: 144.08 g/mol. TLC (5% methanol in dichloromethane), Rf = 0.08.

*2-Fluoroethyl p-toluenesulfonate (2-fluoroethyl tosylate)*

2-Fluoroethyl tosylate was synthesized, as previously described ([3](#_ENREF_3)). Briefly, 2-fluoroethanol (120 mg, 1.87 mmol, Sigma Aldrich) was dissolved, in dry dichloromethane (2 mL), and added to a solution of *p*-toluenesulfonyl chloride (540 mg, 2.83 mmol, Sigma Aldrich), trimethylamine (250 mg, 4.26 mmol, Sigma Aldrich) and DMAP (26 mg, 213 µmol, Sigma Aldrich) in 2 mL dry dichloromethane. The reaction was stirred for 2 h at room temperature. It was subsequently concentrated and purified on a silica gel column, using hexane and ethyl acetate (9:1) as eluent, to yield the title compound. ^1^H NMR (300 MHz, CDCl_3_) δ 2.45 (s, 3H), 4.22 (t, *J*=4.2 Hz, 1H), 4.31 (t, *J*=4.2 Hz, 1H), 4.48 (t, *J*=4.2 Hz, 1H), 4.64 (t, *J*=4.2 Hz, 1H), 7.35 (d, *J*=8.4 Hz, 2H), 7.80 (d, *J*=8.4 Hz, 2H). ^19^F NMR (300 MHz, CDCl_3_) δ -224.65 (h, *J*_1_=0.105, *J*_2_=0.064, 1F).

*N-2-fluoro-ethylquinolinium tosylate*

N-2-fluoro-ethylquinolinium tosylate (FEtQ, Scheme 5s), the non-radiolabeled reference standard for 2-[^18^F]fluoroehtyl quinolinium ([^18^F]FEtQ), was synthesized as described ([4](#_ENREF_4)), with modifications. Briefly, quinoline (175 mg, 1.35 mmol) was added to a solution of 2-fluoroethyl tosylate (1.7 g, 7.8 mmol) in 6 mL dry dimethylformamide (Sigma Aldrich), and the reaction was refluxed for 72 h under an inert atmosphere. The solution was then cooled and concentrated in vacuum to yield a light brown oil. The resulting crude product was purified using an HPLC system equipped with a semi-preparative column (Luna C18, 100 Å, 5 μm, 250×10 mm), using (A) H_2_O: (B) acetonitrile gradient (from 100% to 60% A, over 15 min at a flow rate of 4 mL/min) to yield 215 mg of the trifluoroacetic acid salt (55%). ^1^H NMR (300 MHz, Deuterium Oxide) δ 2.17 (s, 3H), 4.81 (t, *J* = 4.6 Hz, 1H), 4.97 (dd, *J*1 = 4.0 Hz, *J*2 = 5.1 Hz, 1H), 5.19 (t, *J* = 4.6 Hz, 1H), 5.27 (t, *J* = 4.6 Hz, 1H), 7.18 – 7.08 (m, 2H), 7.46 (d, *J* = 8.2 Hz, 2H), 7.93 – 7.78 (m, 2H), 8.07 (ddd, *J*1 = 1.5 Hz*, J*2 = 7.1 Hz, *J*3 = 8.9 Hz, 1H), 9.03 – 8.93 (m, 1H), 8.28 – 8.15 (m, 2H), δ 9.07 (d, *J* = 5.9 Hz, 1H). ^19^F NMR (300 MHz, DMSO-d6) δ -221.14 (m, 1F). HR-MS: calculated: 176.06700; Found: 176.06662.

*Ethylene glycol bistrifluoromethansulfonate*

Ethylene glycol bistriflate was used to prepare the precursor for the radiosynthesis of [^18^F]FEtQ. Its synthesis was carried out as previously described ([5](#_ENREF_5)), with modifications. In brief, a solution of trifluoromethanesulfonic anhydride (1.06 mL, 6.3 mmol, Sigma), in 3 mL of dry dichloromethane (Acros Organics, Geel, Belgium), was stirred on ice under a nitrogen atmosphere and light exclusion. A solution of ethylene glycol (180 µL, 3.2 mmol, Sigma) and dry pyridine (521 µL, 6.4 mmol, Sigma), dissolved in 3.5 mL of dry dichloromethane, was then added over 10 min, after which the solution was left stirring for an additional 15 min. Subsequently, the solution was diluted with 20 mL of dichloromethane and washed twice with cold HPLC water. The organic layer was dried using magnesium sulfate, filtered, and evaporated using an oil pump at 30°C for 30 min, to yield the product as a clear and light red oil (0.85 g, 80.7% yield). ^1^H NMR (300 MHz, CDCl_3_) δ 4.52 (s, 4H). ^19^F NMR (300 MHz, CDCl3) δ -77.10 (m, 6F, *J1* = 27.6 Hz, *J2* = 21.9 Hz).

*2-trifluoromethansulfonateethylquinolinium trifluoromethansulfonate*

A solution of ethylene glycol bistriflate (scheme 6s, 100 mg, 0.55 mmol), in 4 mL of dry dichloromethane, was cooled to -80°C under a nitrogen atmosphere and light exclusion. A solution of quinoline (33 mg, 0.24 mmol, Sigma), in 4 mL dry dichloromethane, was then added over 10 min (Scheme 6s). The solution was allowed to reach room temperature and was left stirring for an additional 48 h. Subsequently, the reaction was cooled in an ice bath for 20 min, and cold ether was added for an additional 20 min. The mixture was filtered through a sintered glass filter equipped with filter paper under reduced pressure, and the precipitate was further washed with 100 mL of cold ether. The collected white precipitate was then dried using dry toluene evaporation to yield the title compound (84.6 mg, 80.4% yield). This reagent was stored at -16°C under nitrogen atmosphere. ^1^H NMR (300 MHz, DMSO-d6) δ 4.77 (t, *J*=4.7 Hz, 2H), δ 5.41 (t, *J*=4.7, 2H), δ 8.0 - 8.36 (m, 4H), δ 8.43 - 8.65 (m, 3H), δ 9.30 – 9.42 (m, 2H). ^19^F NMR (300 MHz, DMSO-d6) δ -77.77 (s, 3F).

*Radiochemistry of fluorinated DMDPA derivatives*

*2-[^18^F]-fluoroethylmethyldiphenylammonium trifluoromethanesulfonate ([^18^F]FEMDPA)*

Cyclotron produced [^18^O]H_2_O/[^18^F^-^] (50 ± 8 GBq, n = 3) was loaded onto an ion-exchange column (pre-activated with 0.8 mL of ethanol and 3 mL of HPLC water), and transferred to a reactor by elution with 0.5 mL of potassium carbonate (6 mg/mL). Then, 15 mg of kryptofix-2.2.2 (ABX, Radeberg, Germany), dissolved in 1 mL acetonitrile (Merck, Darmstadt, Germany), was added. Azeotropic removal of water and acetonitrile was achieved by heating the reactor to 100°C under a stream of nitrogen for 3 min, followed by an additional one min under reduced pressure. Then, diphenylamino 2-methanesulfonic ethyl ester (10 mg, 34 µmol, scheme 7s), dissolved in acetonitrile (1 mL), was added to the reactor, the reactor temperature was increased to 120°C, and the reaction mixture was stirred for 20 min. Volatiles were then removed at a temperature of 120°C, under a stream of nitrogen for one min, and under reduced pressure for additional two min. The reactor was then cooled to 50°C, and 2,6-di-tert-butyl-4-methylpyridine (92 mg, 0.45 mmol), dissolved in dry dichloroethane (400 µL), and methyl trifluoromethanesulfonate, (50 µL, 0.44 mmol) dissolved in dry dichloroethane (200 µL), were added. The reactor temperature was increased to 105°C for 25 min. The solvent was then removed, and the crude product was further diluted, using a solution of ethanol and acetate buffer 0.1 M, pH 3.8 (at a ratio of 7:3). Purification was performed using semi-preparative HPLC equipped with Nucleosil 100-7-C18 reversed-phase semi-preparative column (7 µm, 250X16 mm, Macherey-Nagel, Düren, Germany) at flow rate of 4.8 mL/ min using acetate buffer (0.1 M, pH 3.8) supplemented with 10% ethanol as eluent, yielding 2.7 ± 0.4 GBq of the title compound at 9.4% radiochemical yield (RCY) decay corrected (D.C.) to the end of bombardment (EOB) and a radiochemical purity (RCP) greater than 99%. The identity of the final product was confirmed using an analytical HPLC system, equipped with µ-Bondapack C18 analytical column (10 µm, 3.9x300 mm, Waters, Milford, Ma, USA), a UV detector operated at a wavelength of 254 nm, and a radioactivity detector with NaI crystals. As a mobile phase, a gradient of (A) acetate buffer 0.1 M, pH 3.8 and (B) acetonitrile was used, from 80 % (A) to 20 % (A) over 30 min, at a flow rate of 1.2 mL/min (*[^18^F]FEMDPA* retention time 7 min).

*[^18^F]-4-fluorobutylmethyldiphenylammonium trifluoromethanesulfonate ([^18^F]FBMDPA)*

Cyclotron produced [^18^O]H_2_O/[^18^F^-^] was loaded onto ion-exchange column (pre-activated with 0.8 mL of ethanol and 3 mL of HPLC water) and transferred to a reactor by elution with 0.5 mL of potassium carbonate (3.3 mg/mL). Then, a solution of kryptofix-2.2.2 (10 mg) dissolved in 1 mL acetonitrile was added to the reactor, followed by azeotropic removal of water and acetonitrile by heating the reactor to 100ºC under a stream of nitrogen for six min, and under reduced pressure for additional two min. Subsequently, 4-iodobutyl diphenylamine (20 mg, 56.8 μmol), dissolved in 1 mL of acetonitrile, was added to the reactor, after which it was sealed and heated to 105ºC for 10 min, yielding the intermediate [^18^F]-4-fluorobutyl diphenylamine (Scheme 8s). The identification of the intermediate compound was confirmed by co-elution with the non-radiolabeled reference standard, using analytical HPLC. Then, methylation of [^18^F]-4-fluorobutyl diphenylamine was performed using methyl trifluoromethanesulfonate (1.37 mmol), dissolved in 800 µL anhydrous dichloroethane, in a sealed and heated reactor to 120ºC for 10 min, followed by cooling to 40ºC. The reaction was then diluted, using 2 mL solution of HPLC H_2_O and acetonitrile (1:1) supplemented with 0.1 % trifluoroacetic acid, and was purified using a semi-preparative HPLC system equiped reverse phase C18 column (Nucleosil 100-7 C-18, 250x16mm, Macherey-Nagel, Düren, Germany). As a mobile phase, (A) HPLC H_2_O and (B) acetonitrile, both supplemented with 0.1 % trifluoroacetic acid, were used, with a gradient starting from 80% (A) to 20% (A) over 30 min, at flow rate of 6 mL/ min. The final product (retention time 8 min.), obtained with an overall RCY of 4.3 ± 1.9% (n = 5) D.C. to EOB, a RCP of 99%, and mean molar activity of 109 ± 56 GBq/µmol, was further diluted with 0.9 % sodium chlorides soluion for injection. The identity of the final product was confirmed by co-elution with the reference standard using an analytical HPLC system, equipped with µ-Bondapack C18 analytical column (10 µm, 3.9x300 mm, Waters, Milford, Ma, USA) at a flow rate of 1 mL/ min, a UV detector operated at a wavelength of 254 nm, and a radioactivity detector with NaI crystals. As mobile phase, a gradient of (A) acetate buffer 0.1 M, pH 3.8 and (B) acetonitrile was used, from 80 % (A) to 20 % (A) over 30 min ([^18^F]FBMDPA retention time = 18 min). The title compound and free ^18^F^-^ were also detected by radio-TLC, using silica gel plates (Merck, Darmstadt, Germany) and 5% methanol in dichloromethane as mobile phase (R.F. = 0.6).

*[^11^C]methyl-(3-fluorophenyl)methylphenylammonium trifluoromethanesulfonate ([^11^C]3-F-DMDPA) and [^11^C]methyl-(4-fluorophenyl)methylphenylammonium trifluoromethanesulfonate ([^11^C]4-F-DMDPA)*

The two ^11^C-labeled, ^19^F-fluorinated derivatives of [^11^C]DMDPA ([^11^C]3-F-DMDPA and [^11^C]4-F-DMDPA, Figure 1) were prepared, as previously described for [^11^C]DMDPA ([1](#_ENREF_1)), using the respective ^19^F-fluorinated methyldipenylamines as precursors.

*Radiochemistry of quinoline-based analogs*

*[^11^C]-methylquinolinium iodide ([^11^C]MeQ)*

Carbon-11 CO_2_ (31.5 ± 4.8 GBq, n = 3) was trapped at -160°C. The temperature of the cooling trap was then increased to -20°C, and the activity was transferred by a stream of argon (40 mL/min) into the first reactor, containing 300 μL of 0.25 M lithium aluminum hydride in tetrahydrofuran (ABX, Radeberg, Germany), at -50°C. After 2.5 min, the solvent was removed under reduced pressure, and the reactor temperature was increased to 160°C. Subsequently, hydroiodic acid (Merck, Darmstadt, Germany) was added, and [^11^C]CH_3_I was distilled through a NaOH column (Merck, Darmstadt, Germany) using argon flow (25 mL/min). The resulting [^11^C]CH_3_I was transferred into a second reactor containing quinoline (30 mg, 0.23 mmol), dissolved in 400 μL dry acetonitrile at -20°C. At the end of the one-min distillation step, 19 ± 1 GBq (n = 3) was trapped in the second reactor, which was sealed, and heated to 80°C for 7 min. Then, the solvent was removed under argon flow at 75°C. The mixture was cooled to 40°C, followed by the addition of a 2 mL solution of water and ethanol (1:1), and the crude product was transferred into a flask containing 5 mL of water. Subsequently, the crude product was loaded onto an SPE cartridge (Sep-Pak Accell Plus CM light cartridge, Waters, Milford, Ma, USA, pre-activated using 20 mL HPLC water). The cartridge was then further washed with 5 mL of water, and the final product was eluted using 5 mL of sterile isotonic saline (0.9% sodium chloride solution, B. Braun, Melsungen, Germany) and filter sterilized using a 0.22 µm filter (Cathivex-GV, Darmstadt, Germany). The identification of the product was confirmed by co-elution with the reference standard, using analytical HPLC equipped with a Luna C18 RP-C18 column (100Å, 5 µm, 250× 10 mm, Phenomenex, Torrance, CA, USA), a UV detector at a wavelength of 315 nm, and a radioactivity detector with NaI crystals. The HPLC mobile phase was (A) HPLC H_2_O and (B) acetonitrile, both suplemnetd with 0.05% TFA, at a flow rate of 1 mL/ min using a gradient from 95 % (A) to 15 % (A) during 17 minutes. Overall, following a total radiosynthesis and purification time of 30 min, 2.7 ± 0.7 GBq of [^11^C]-N-methylquinolinium iodide was obtained (n = 3, scheme 9s) with 26 ± 3% radiochemical yield, D.C. to the EOB. RCP was routinely greater than 95%, and the molar activity greater than 37 GBq/μmol, D.C. to EOS.

*[^18^F]-fluoroethylquinolinium acetate* *([^18^F]FEtQ)*

Cyclotron produced [^18^O]H_2_O/[^18^F^-^] (131.3 ± 12.5 GBq, n = 13) was loaded onto ion-exchange column (pre-activated with 0.8 mL of ethanol and 3 mL of HPLC water), and transferred to a reactor by elution, using 0.5 mL of potassium carbonate (8 mg/mL) followed by addition of kryptofix-2.2.2 (15 mg), dissolved in 1 mL acetonitrile. Azeotropic distillation was performed under steam of argon and heating to 95 °C for 5 min, followed by an additional 3 min of reduced pressure (5 kpa), followed by cooling to 50°C. Then, ethyltrifluoromethanesulfonate quinolinium (15 mg), dissolved in 1.4 mL dry dichloromethane (acros organics, Geel, Belgium), was added to the reactor and the fluorination step was performed in a sealed reactor at 95°C for 5 min (Scheme 10s). The reaction mixture was then cooled, and diluted with 1.9 mL of acetate buffer pH 5.2, 0.1 M supplemented with 3% ethanol. The crude product mixture was filtered through a polypropylene 0.45 µm filter (Whatman, G.E. Healthcare, Chicago, USA) and further purified using a semi-preparative HPLC system, equipped with Luna C18 RP-C18 column (100Å, 5 µm, 250× 10 mm, Phenomenex, Torrance, CA, USA), and acetate buffer pH 5.2, 0.1 M and ethanol (97:3) as eluent at a flow rate of 4 mL/min. [^18^F]FEtQ retention time was 18.5 min, and the final product (2-4 mL) was collected in a product vial containing 5 mL of 0.9% sodium chloride solution for injection. Chemical and radiochemical purities were determined using an analytical HPLC, equipped with a UV detector at 316 nm. Overall, following a total synthesis time of 44 min, 13.3 ± 4.3 GBq of [^18^F]FEtQ was obtained with an average radiochemical yield of 14.2 ± 4%, after purification, D.C. to EOB. RCP was routinely greater than 95%, and the mean molar activity was 55.3 ± 5 GBq/μmol, D.C. to EOS.

**Schemes**:

**Scheme 1s:** Synthesis of 2-fluoroethyl methyldiphenylamine trifluoromethanesulfonate (FEMDPA). (a) NaH, n-buthyl ether, 135°C, 3 h (b) 1-bromo-2-fluoroethyl, 0-120°C, 3 h (c) methyl trifluoromethansulfonate, dichloroethane, reflux, 3 h.

**Scheme 2s:** Synthesis of 4-fluorobuthyl methyldiphenylamine trifluoromethanesulfonate (4-FBMDPA). (a) NaH, THF, 1-iodo-4-fluorobutane, reflux, 16 h (b) methyl trifluoromethansulfonate dichloroethane, 0°C, 3 h.

**Scheme 3s:** Synthesis of DMDPA trifluoromethanesulfonate (X_1_ = X_2_ = H), 3-F-DMDPA trifluoromethanesulfonate (X_1_ = H, X_2_ = F) and 4-F-DMDPA trifluoromethanesulfonate (X_1_ = F, X_2_ = H). (a) Methyl trifluoromethansulfonate, dichloroethane, reflux 6 h. (b) methyl trifluoromethansulfonate, dichloroethane, reflux 6 h.

**Scheme 4s:** Synthesis of 1-methylquinolinium iodide. (a) Methyl iodide, 40°C, 3 h.

**Scheme 5s:** Synthesis of N-2-fluoroethylquinolinium tosylate (FEtQ)**.** (a) 2-Fluoroethyl tosylate, dimethylformamide, 100°C, 48 h.

**Scheme 6s:** Synthesis of 2-trifluoromethansulfonate ethylquinolinium trifluoromethansulfonate. (a)Trifluoromethanesulfonic anhydride, dimethylformamide, -70°C –20°C, 48 h.

Scheme 7s: Synthesis of [^18^F]fluoroethyl methyldiphenylammonium trifluoromethansulfonate ([^18^F]FEMDPA). (a) K^18^F, Kryptofix-2.2.2., acetonitrile, 90°C, 20 min (b) methyl trifluoromethansulfonate, dichloroethane, 90°C, 20 min.

Scheme 8s: Synthesis of [^18^F]fluorobutyl methyldiphenylammonium trifluoromethansulfonate ([^18^F]FBMDPA). (a) K^18^F, Kryptofix-2.2.2., acetonitrile, 105°C, 10 min (b) methyl trifluoromethansulfonate, dichloroethane, 120°C, 10 min.

Scheme 9s: Synthesis of [^11^C]methylquinolinium iodide ([^11^C]MeQ). (a) [^11^C]CH_3_I, acetonitrile, distilled at -20°C followed by heating to 80°C for 7 min.

Scheme 10s: Synthesis of [^18^F]fluoroethylquinolinium acetate ([^18^F]FEtQ). (a) K^18^F, Kryptofix-2.2.2., dichloromethane, 95°C, 15 min

*References:*

1. Ilovich, O., Abourbeh, G., Bocher, M., Freedman, N., Billauer, H., Dotan, S., Danenberg, H. D., and Mishani, E. (2012) Structure--activity relationship and preclinical evaluation of carbon-11-labeled ammonium salts as PET--myocardial perfusion imaging agents. *Mol. Imaging Biol.* **14**, 625-636

2. Jacobson, O., Abourbeh, G., Tsvirkun, D., and Mishani, E. (2013) Rat imaging and in vivo stability studies using [11C]-dimethyl-diphenyl ammonium, a candidate agent for PET-myocardial perfusion imaging. *Nucl. Med. Biol.* **40**, 967-973

3. Damont, A., Hinnen, F., Kuhnast, B., Schöllhorn-Peyronneau, M., James, M., Luus, C., Tavitian, B., Kassiou, M., and Dollé, F. (2008) Radiosynthesis of [18F]DPA-714, a selective radioligand for imaging the translocator protein (18 kDa) with PET. *J. Label Compd. Radiopharm* **51**, 286-292

4. Parenty, A. D. C., Smith, L. V., and Cronin, L. (2005) An unusual substitution reaction directed by an intramolecular re-arrangement. *Tetrahedron* **61**, 8410-8418

5. Stewart, J. C. M. (2012) Dipyridinium derivatives. Google Patents
